# Atmospheric Exposure Triggers Light-Induced Degradation in 2D Lead-Halide Perovskites

**DOI:** 10.1021/acsenergylett.4c02300

**Published:** 2024-11-07

**Authors:** Gianluca Grimaldi, Imme Schuringa, Jaco J. Geuchies, Susan A. Rigter, Tom Hoekstra, Jan Versluis, Juanita Hidalgo, Juan-Pablo Correa-Baena, Jorik van de Groep, Heejae Kim, Mischa Bonn, Bruno Ehrler

**Affiliations:** † Center for Nanophotonics, AMOLF, Science Park 104, 1098 XG Amsterdam, The Netherlands; ‡ Optoelectronics Section, Cavendish Laboratory, University of Cambridge, Cambridge CB2 1TN, U.K.; § Department of Molecular Spectroscopy, Max Planck Institute for Polymer Research, 55128 Mainz, Germany; ∥ Van der Waals-Zeeman Institute, Institute of Physics, University of Amsterdam, Science Park 904, Amsterdam 1098 XH, The Netherlands; ⊥ School of Materials Science and Engineering, Georgia Institute of Technology, Atlanta, Georgia 30332, United States

## Abstract

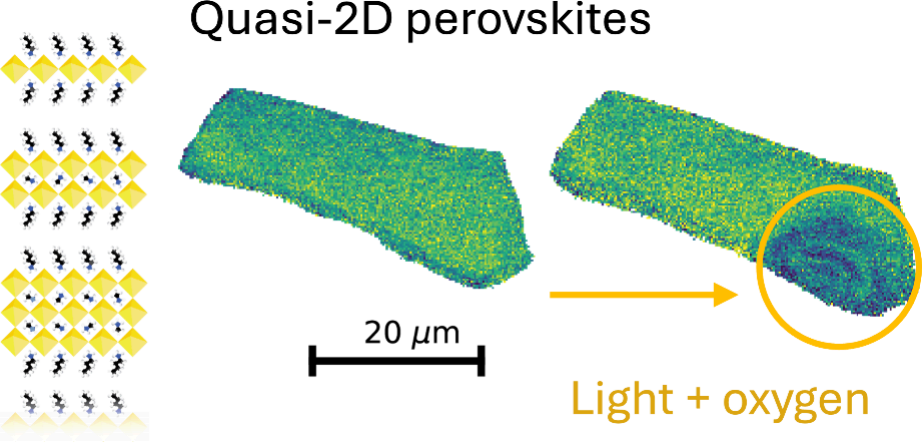

Quasi-2D perovskites have been pivotal in recent efforts to stabilize perovskite solar cells. Despite the stability boost provided when these materials are introduced in perovskite solar cells, little is known about the intrinsic light and environmental stability of quasi-2D perovskites. In this study, we characterize the photostability of exfoliated quasi-2D perovskite single crystals in air using photoluminescence, infrared, X-ray fluorescence, and energy-dispersive X-ray spectroscopy. Photoexcitation leads to severe material loss with oxygen as a prerequisite for material breakdown. The effect can be traced to the formation of reactive oxygen species, as demonstrated by increases in the photostability under oxygen-free conditions. We show the effect of combined passivation steps, showcasing the stability enhancement offered by 2D-capping layers in combination with an oxygen-free atmosphere. Our results reveal that the stability of illuminated quasi-2D perovskites depends critically on oxygen exposure, highlighting the importance of oxygen-blocking passivation strategies for stable 2D perovskite-based devices.

The rapid advance in perovskite photovoltaics has pushed this technology from impractical proof-of-concept devices to the brink of commercialization in the span of a decade.^1^ Perovskite solar cells can now be fabricated with efficiencies comparable to those of single-crystalline silicon solar cells, shifting the technological bottleneck away from performance and toward stability. In the search for more stable perovskite compositions, low-dimensional perovskites have been identified as an attractive material candidate, and solar cell stacks featuring low-dimensional perovskites alongside conventional (3D) perovskites have demonstrated a remarkable combination of performance and stability. In particular, the 2D Ruddlesden–Popper phases, commonly known as two-dimensional (2D) perovskites, have been the focus of extensive research in the last couple of years, as incorporating a layer of 2D perovskites between a 3D perovskite light-absorber and a transport layer can lead to >20% efficiency solar cells with an operational lifetime of over 1000 h.^2−8^

The rationale behind the stabilization effect can be found in the structural properties of 2D perovskites, characterized by 2D layers of lead halide octahedra separated by layers of long organic molecules, called spacers. These materials can be classified by the number *n* of octahedral monolayers sandwiched between two spacer layers, where *n* = 1 is often referred to as a 2D perovskite proper and 1 < *n* < 10 is often called quasi-2D perovskite^9^ (see Figure 1a). Perovskites with different *n*-values display substantial variations in electronic and optical properties,^10,11^ due to quantum- and dielectric-confinement of the electronic wave functions in the 2D layers. These materials show increased hydrophobicity compared to their 3D counterparts, which is hypothesized to enhance their resistance to moisture-induced degradation processes prevalent in 3D perovskites.^2,12,13^ Additionally, evidence of reduced ionic mobility in (quasi-) 2D perovskites in the dark^14,15^ and under illumination^16,17^ suggest incorporating these materials in a perovskite solar cell might mitigate degradation processes triggered by ion migration, such as ionic reactions with contacts and transport layers.

**Figure 1:**
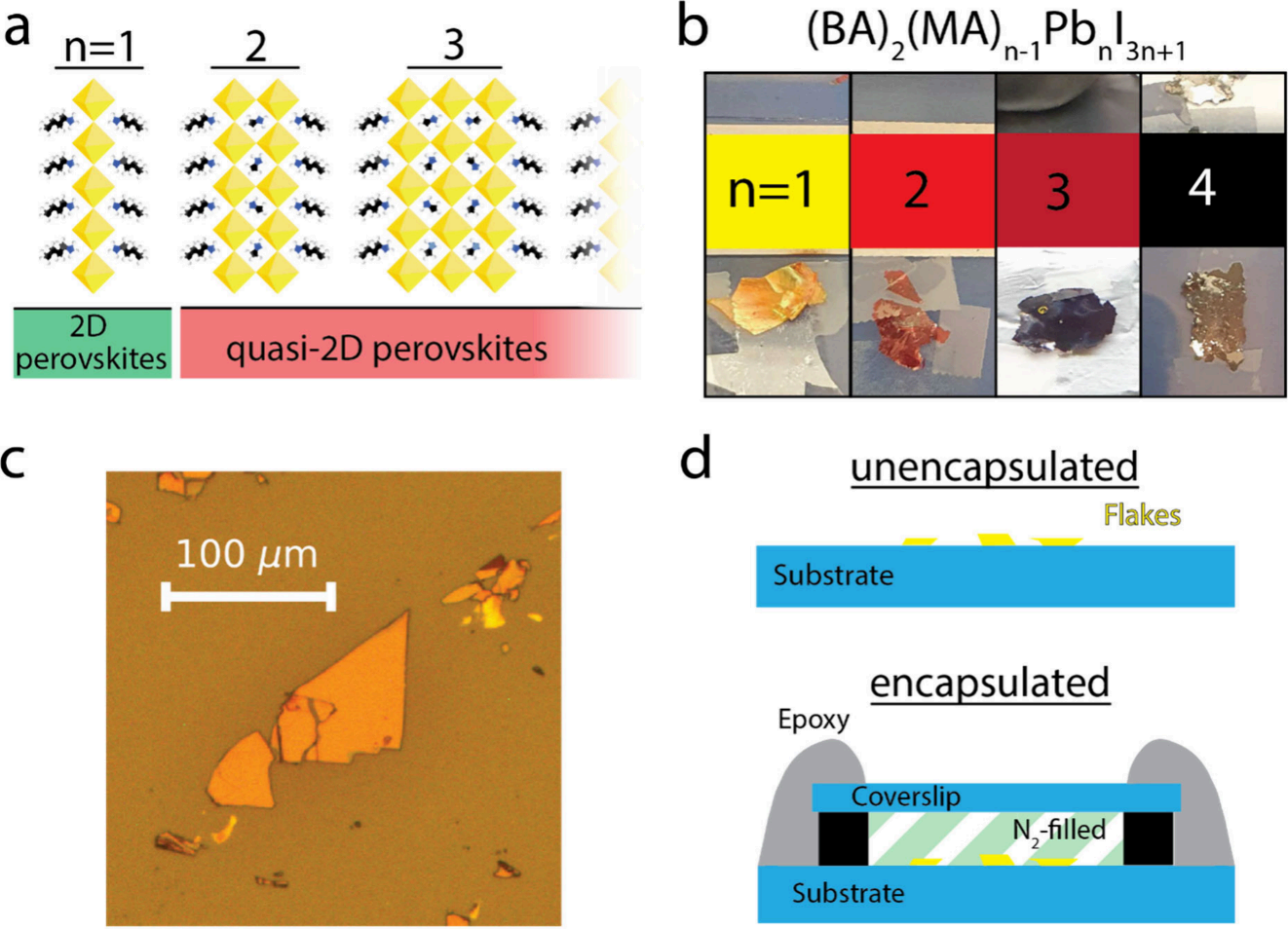
Preparation of (quasi-) 2D perovskite samples. (a) Schematics depicting the crystal structure of (BA)_2_(MA)_*n*−1_Pb_*n*_I_3*n*+1_. (b) Pictures of the (quasi-) 2D perovskite crystals used in this work. (c) Optical microscope image of exfoliated *n* = 2 flakes. (d) Schematics representing the sample geometry with and without the encapsulation step.

These properties are prompting researchers in the perovskite solar cell community to consider (quasi-) 2D perovskites as stable alternatives to 3D perovskites. However, recent reports have cast doubts on the degree of stability of (quasi-) 2D perovskites exposed to heat,^18^ intense illumination,^19,20^ and vacuum,^21^ conditions relevant for solar cell operation or fabrication. In particular, Fang et al. have highlighted the rapid photodegradation process affecting single crystals of (PEA)_2_PbI_4_, an *n* = 1 2D perovskite. Upon illumination, the crystals undergo morphological and photoluminescence changes, attributed to the degradation of the 2D perovskite and the formation of PbI_2_.^19^

Despite these warning signs, little is known about the intrinsic photostability of *n* > 1 quasi-2D perovskites, the versions of the material predominantly present in solar cell architectures. Furthermore, while applying passivation layers on top of *n* = 1 2D perovskite crystals has been shown to increase their photostability,^19,22^ the effect has been attributed to a reduction in photoinduced desorption of volatile species from the crystals,^19^ without clarifying the role of airborne species (i.e., O_2_, H_2_O). Disentangling the impact of quasi-2D perovskite photostability from the complex system of electronic and ionic processes affecting 2D/3D solar cells is necessary to understand stability trends in these devices and to guide the design of stable perovskite solar cell architectures.

In this study, we investigate the stability of single crystals of (BA)_2_(MA)_*n*−1_Pb_*n*_I_3*n*+1_ (quasi-) 2D perovskites (*n* = 1, ..., 4) under laser illumination. We monitor local morphological and optical changes using a combination of photoluminescence (PL) microscopy, time-resolved PL imaging, optical microscopy, scanning electron microscopy-based energy dispersive X-ray (SEM/EDX) spectroscopy, Fourier-transform infrared (FTIR) spectroscopy, and X-ray fluorescence (XRF) imaging. Our results show that quasi-2D perovskites suffer from rapid photodegradation processes comparable to those affecting the *n* = 1 2D perovskite species, leading to PL decay and material loss in the photoexcited spots. To elucidate the degradation mechanism, we monitored the evolution of the crystals exposed to different atmospheres, revealing the crucial role of oxygen in facilitating morphological and photoluminescence changes. Illuminating in the absence of O_2_ prevents morphology changes entirely in *n* > 1 quasi-2D perovskites, leading to milder PL losses attributed to the formation of electronic traps. Finally, we show that a combination of an O_2_-free atmosphere and applying a 2D capping layer increases the photostability, suggesting the presence of both intrinsic (volatile species desorption) and extrinsic (O_2_-induced) degradation pathways.

In order to obtain flakes of (quasi-) 2D perovskite materials, we synthesized centimeter-sized crystals of (BA)_2_(MA)_*n–*1_Pb_*n*_I_3*n*+1_ (BA = butylammonium, MA = methylammonium), with *n* = 1, ..., 4 (see Figure 1b). The material is pure-phase (only the nominal *n*-value present) for *n* = 1, and photoluminescence spectra from *n* = 2–4 are dominated by the nominal *n*-value (see Figure S1, Supporting Information). The large crystals were mechanically exfoliated in a nitrogen-filled glovebox using a tape-based exfoliation method commonly used for van der Waals 2D materials and then stamped on a glass substrate. The procedure resulted in the transfer of thin (a few hundred nanometers thick) flakes on the substrate, with lateral dimensions extending for tens of micrometers (see Figure 1c). The large lateral extension of the flakes allows the photoexcitation of selected spots on a single flake with a PL microscope, enabling direct comparison of the morphological and compositional characteristics of photoexcited spots with those of adjacent unexcited areas.

To determine the impact of air on the photostability of (quasi-) 2D perovskites, we prepare two sets of samples: one prepared via the exfoliating/stamping procedure described above and the second featuring an additional encapsulation step performed in the glovebox. The encapsulation allows optical measurements outside of the glovebox while maintaining the flakes in an O_2_- and H_2_O-free atmosphere. To encapsulate the samples, we place a 170 μm thick glass coverslip on top of the area with the stamped crystals, and we separate it from the substrate by strips of carbon tape. The edges of the coverslip are then covered with UV-cured epoxy, sealing it to the substrate (see Figure 1d). The resulting airtight enclosure is filled with nitrogen to assess degradation without air and water, or with dry air to isolate the effect of O_2_. This last sample also helps to distinguish between protection by the glass capping layer and removal of the O_2_.

To characterize the stability of the unencapsulated flakes, we exposed flakes with different *n*-values to 405 nm laser light in a photoluminescence (PL) microscopy setup, allowing local photoexcitation of 15 μm wide spots on the flakes, while measuring the evolution of the PL during the exposure period. We exposed multiple spots on each flake using a fixed exposure time (∼180 s) and varying laser fluence (spanning the range from 2.2 × 10^18^ to 4.5 × 10^19^ photons/(s cm^2^)), to investigate the dependence of photodegradation effects on the incident energy dose. Figure 2a shows optical microscopy images of *n* = 1 to *n* = 4 flakes, comparing their morphology before and after localized photoexcitation (photoexcited spots highlighted with dashed circles). All flakes show signs of local morphology changes in the photoexcited spots, revealed by the appearance of a clear contrast compared to the unexposed part of the flakes. We note a color change of the flakes next to the excited spots on the *n* = 1 and *n* = 2 flakes, which we tentatively attribute to surface reactions with volatile species emitted from the photoexcited areas, as the effect seems to extend to neighboring flakes (Figure S2). Nevertheless, the excitation spots show the most prominent color changes for all of the *n*-values, suggesting local compositional changes. Since the effect is observed even at the lowest fluence measured, with a photon flux 4 times the average AM1.5 value,^23^ it has the potential to affect quasi-2D perovskite solar cells in high-irradiance conditions.

**Figure 2:**
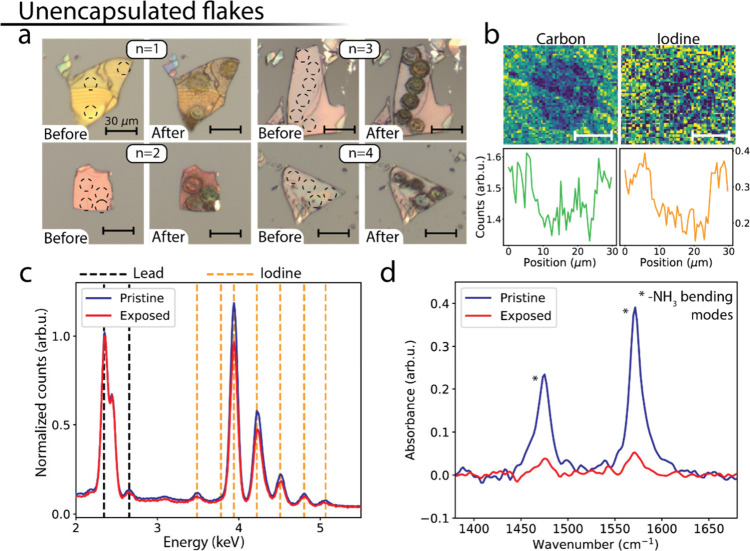
Morphology and compositional changes in unencapsulated flakes. (a) Optical microscope images of *n* = 1, ..., 4 flakes before and after local photoexcitation, showing the presence of color changes in the photoexcited spots. (b) EDX maps of a photoexcited spot in an *n* = 2 flake, displaying the spatial variations in the carbon (top-left) and iodine signals (top-right). The scale bars are 10 μm wide. The bottom panels show a horizontal crosscut of the signal shown in the top panels. (c) Spectrally resolved EDX signal, spatially integrated over the exposed area shown in (b) and over a pristine area of the same flake. (d) FTIR signal measured on an exposed and a pristine area of an *n* = 1 flake, shown in the spectral range containing peaks associated with BA vibrational modes.

To investigate changes in the composition of the flakes following photoexcitation, we performed scanning electron microscopy/energy dispersive X-ray (SEM/EDX) measurements on photoexcited unencapsulated flakes. Figure 2b shows an EDX map of an *n* = 2 flake, with the EDX signal integrated over a carbon peak (top-left panel) and an iodine peak (top-right panel), while the bottom panels show horizontal cross-cuts of the signal for each map. Both images show a decrease in signal intensity in correspondence with the photoexcited spot, suggesting that the morphology changes observed in optical images occur with a significant loss of iodine and organic components. An increase in the lead signal in the photoexcited area is also observed (Figure S3), consistent with a higher lead density in the decomposition product. Figure 2c shows an overview of the lead and iodine peaks in the EDX spectrum integrated over the exposed and pristine areas of the flake, normalized by the intensity of the highest lead peak to reveal variations in the I/Pb ratio. X-ray fluorescence (XRF) measurements on a photoexcited *n* = 3 flake show a similar reduction of the iodine component (Figure S4). The decrease in the I-peak intensities in the photoexcited area reveals a relative loss of iodine compared to the lead component, indicating the formation of volatile iodine species upon photoexcitation. Similar volatile iodine species have been reported in analogous systems.^19,21^ We performed FTIR measurements to monitor changes in the organic components found in the photoexcited spots. Figure 2d shows FTIR measurements obtained on the exposed and pristine region of an *n* = 1 flake, focusing on the presence of sharp peaks in a spectral region compatible with vibrational modes of the BA spacer cation (Figures S5–S7). The intensity of the peaks is strongly suppressed after photoexcitation, indicating a high degree of organic component loss in the photoexcited areas. Despite the low sensitivity of EDX measurements to low atomic number components like carbon, a similar decrease is observed for the C/Pb ratio in the EDX measurements (Figure S3b).

These morphological and compositional observations on *n* = 1 and *n* > 1 flakes are compatible with a degradation mechanism proposed for phenethylammonium-based *n* = 1 2D perovskites,^19,21^ involving the conversion of illuminated 2D perovskite flakes into PbI_2_, accompanied by the formation of volatile iodine and carbon byproducts. In the proposed reaction, the illuminated 2D perovskite decomposes without the involvement of external species, and the stability improvement observed when stacking a protecting material on top of the flakes was attributed to decreased desorption of the volatile reaction products.^19^ However, the impact of O_2_ and H_2_O on the dynamics and nature of the degradation has not yet been assessed, despite the crucial role these species play in the photostability of 3D perovskites.^24−27^

To ascertain the role played by ambient exposure in the degradation process, we repeated for the encapsulated samples the same measurements discussed above. These measurements allow for the photoexcitation of the flakes in a nitrogen atmosphere without restricting the evaporation of volatile degradation products. Figure 3a shows optical images of (quasi-) 2D perovskite flakes taken before and after local photoexcitation (laser fluences in the same range used in Figure 2). Looking at the comparison, it is evident that encapsulation drastically reduces the photoinduced morphological changes. While subtle contrast changes in the photoexcited spots remain apparent for the *n* = 1 flake, *n* > 1 quasi-2D perovskite flakes do not show morphology changes, nor is there evidence of the long-range changes that we observe in the unencapsulated *n* = 1 and *n* = 2 samples. No evidence of compositional change is observed in spatially (Figure 3b) and spectrally resolved (Figure 3c) EDX measurements on exposed and pristine areas of an *n* = 2 flake. Figure 3d shows the FTIR peaks associated with BA vibrational modes in an *n* = 1 flake, revealing a negligible signal change between a pristine area and an area photoexcited under N_2_ atmosphere. A similar lack of changes in the FTIR peaks associated with BA vibrational modes is observed in quasi-2D *n* = 2 flakes photoexcited under an N_2_ atmosphere (Figure S6). Our results demonstrate that the presence of air plays an active role in the photodegradation process. Additionally, a nitrogen atmosphere leads to complete prevention of material losses in *n* > 1 flakes upon photoexcitation.

**Figure 3:**
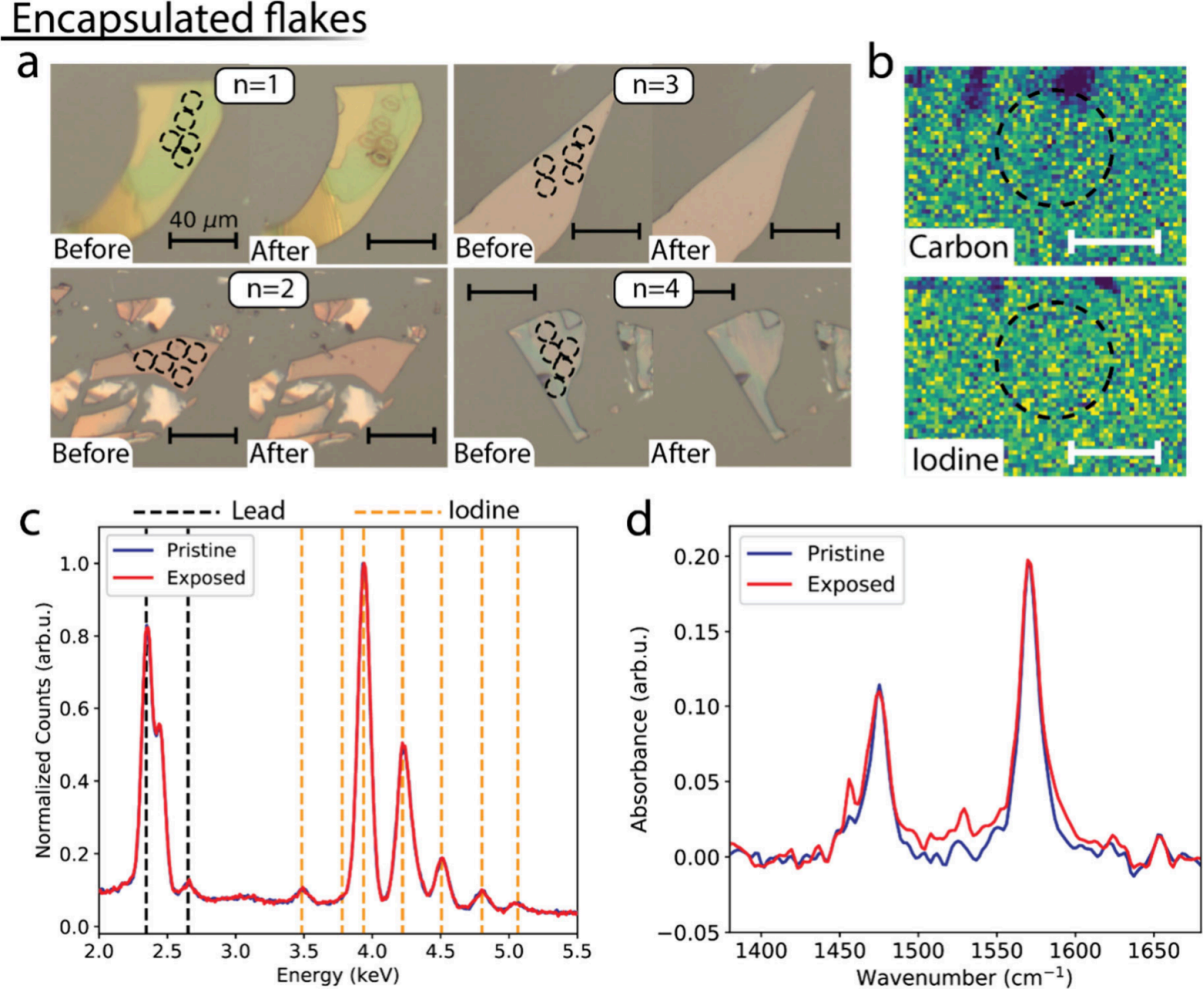
Morphology and compositional changes in encapsulated flakes. (a) Optical microscope images of *n* = 1, ..., 4 flakes before and after local photoexcitation, showing reduced morphology changes in the *n* = 1 flake and no change in the *n* > 1 flakes. (b) EDX maps of a photoexcited spot in an *n* = 2 flake, showing a lack of spatial variations in the carbon (top) and iodine signals (bottom) between exposed and pristine areas. The scale bars are 7 μm wide. (c) Spectrally resolved EDX signal, spatially integrated over the exposed area shown in (b) and over a pristine area of the same flake. (d) FTIR signal measured on an exposed and a pristine area of an *n* = 1 flake photoexcited in a N_2_ atmosphere.

While atmospheric control leads to strong suppression of the morphological and compositional changes, its impact on the optical property changes after photoexcitation appears more nuanced. Figures 4a–d shows the PL intensity obtained during laser exposure of *n* = 1 to *n* = 4 flakes, plotted as a function of the radiation energy dose received during exposure (i.e., exposure time multiplied by the incident power). The traces for the encapsulated samples (continuous lines) reveal that, for all *n*-values and all laser powers used, the PL signal decays as a function of the incident dose, indicating that the encapsulation does not fully prevent material degradation under illumination. We point out the presence of an initial PL increase at low incident doses for the unencapsulated *n* > 1 flakes, likely associated with the passivating effect of oxygen molecules binding to iodide vacancies reported in 3D perovskites.^28,29^

**Figure 4:**
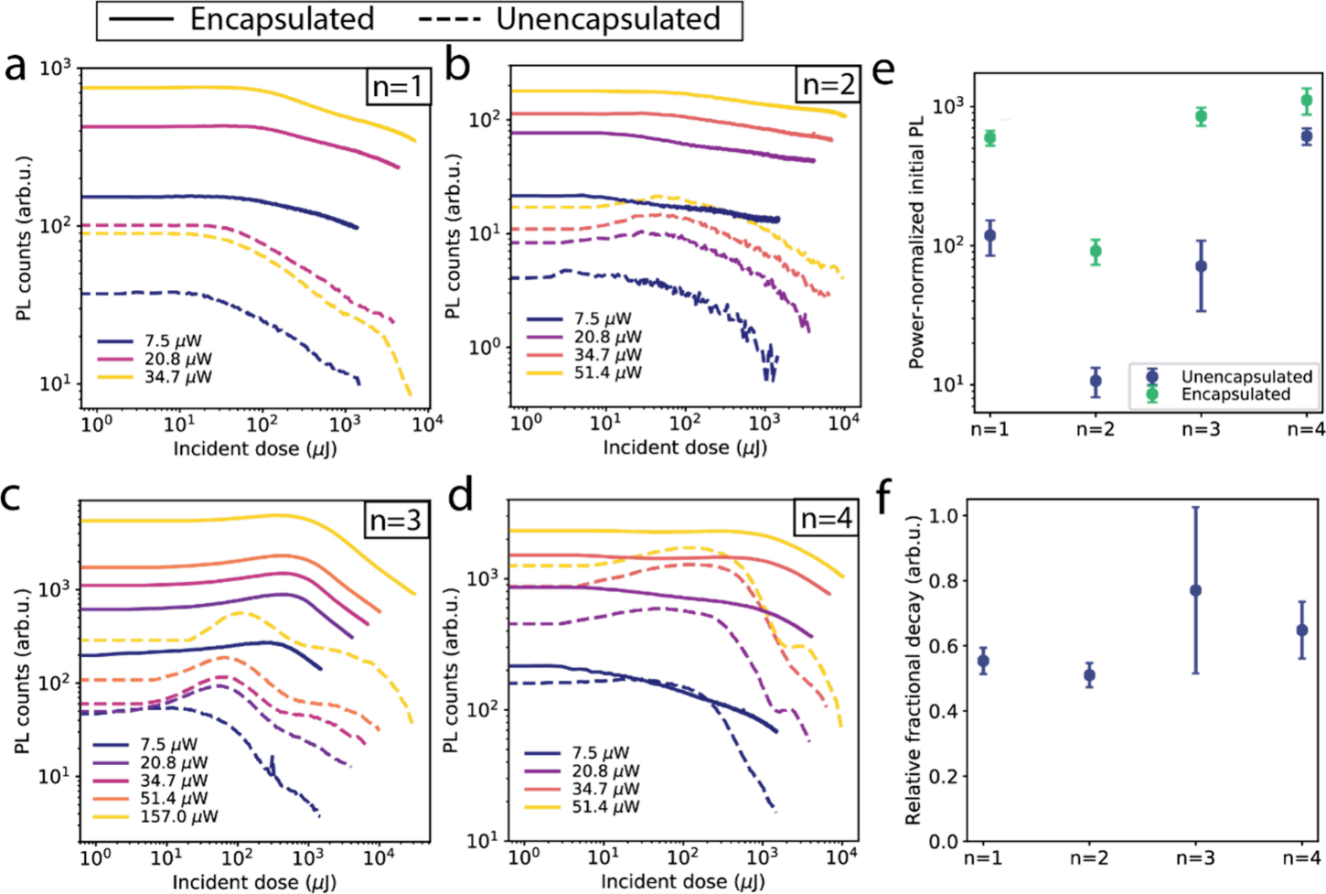
Impact of light-induced degradation on the PL of encapsulated and unencapsulated flakes. (a–d) PL intensity plotted as a function of the incident radiation dose (product of the laser power and the exposure time), for increasing laser power (darker to lighter) for encapsulated (continuous line) and unencapsulated samples (dashed line). (e) Intensity of the photoluminescence at the beginning of the exposure window, normalized by the laser power. Comparing values for the encapsulated and unencapsulated samples reveals an increased PL efficiency in the encapsulated samples. (f) Plot of the relative fractional decay, *F*_E_/*F*_U_, for the different *n*-values. The ratio remains below 1 for all *n*-values, indicating slower degradation dynamics in the encapsulated samples.

Despite the presence of photodegradation in the encapsulated samples, a comparison with the unencapsulated samples (dashed lines) highlights the beneficial effect of encapsulation on the initial intensity of the PL signal. Figure 4e shows the initial PL intensity normalized by the laser power and averaged over the measurements with different power. The plot reveals that, regardless of the power used, encapsulation considerably improves the initial PL intensity, likely due to air-induced increase in nonradiative recombination occurring before the measurement.^22^

Furthermore, the encapsulation helps slow the rate of PL decay. To quantify this effect, we define the fractional PL decay as
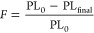
1where PL_0_ indicates the initial PL intensity and PL_final_ is the PL intensity at the highest dose. This quantity represents the fractional loss of PL at the end of the exposure and can be used as an indicator of the severity of the degradation of PL intensity during the exposure time. We can then compare the degradation of encapsulated and unencapsulated samples by considering the ratio of the fractional decays *F*_E_/*F*_U_, where the E and U subscripts refer to the encapsulated and unencapsulated samples, respectively. Figure 4f shows *F*_E_/*F*_U_ for the different *n*-values, with each data point averaged over measurements with different fluences (Figure S8). The ratio is below 1 for all *n*-values, indicating a decreased rate of degradation for the encapsulated flakes. Overall, the PL dynamics under illumination reveal that encapsulation, while slowing down the dynamics of PL losses, does not fully prevent degradation of the electronic properties of the (quasi-) 2D perovskite flakes.

In the absence of a clear material loss mechanism, photoluminescence quenching is associated with increased nonradiative recombination. To investigate this effect locally, we measured time-resolved PL, monitoring for changes in the emission lifetime between exposed and pristine areas of the flakes (see Methods and Figure S9 in the Supporting Information for the description of the fit). Figure 5a,b shows optical and PL lifetime maps of the same n = 1 flake, both before (left) and after exposure (right). Even though the optical images do not show clear signs of morphological changes, the lifetime maps show the appearance of a region that exhibits a shorter lifetime after photoexcitation. A similar lifetime shortening is observed in Figure 5c, showing the time dependence of the PL integrated over the exposed spot and the pristine area. Fitting the data with a single exponential decay (Figure S10, Supporting Information) gives a lifetime of 431.0 ± 0.2 ps on the pristine area and 388.9 ± 0.2 ps in the exposed area. Since the PL decay is determined by the competition between radiative and nonradiative recombination, a shortening of the decay lifetime accompanied by a decrease in PL intensity indicates an increase in nonradiative recombination in the morphologically unaltered spots.

**Figure 5:**
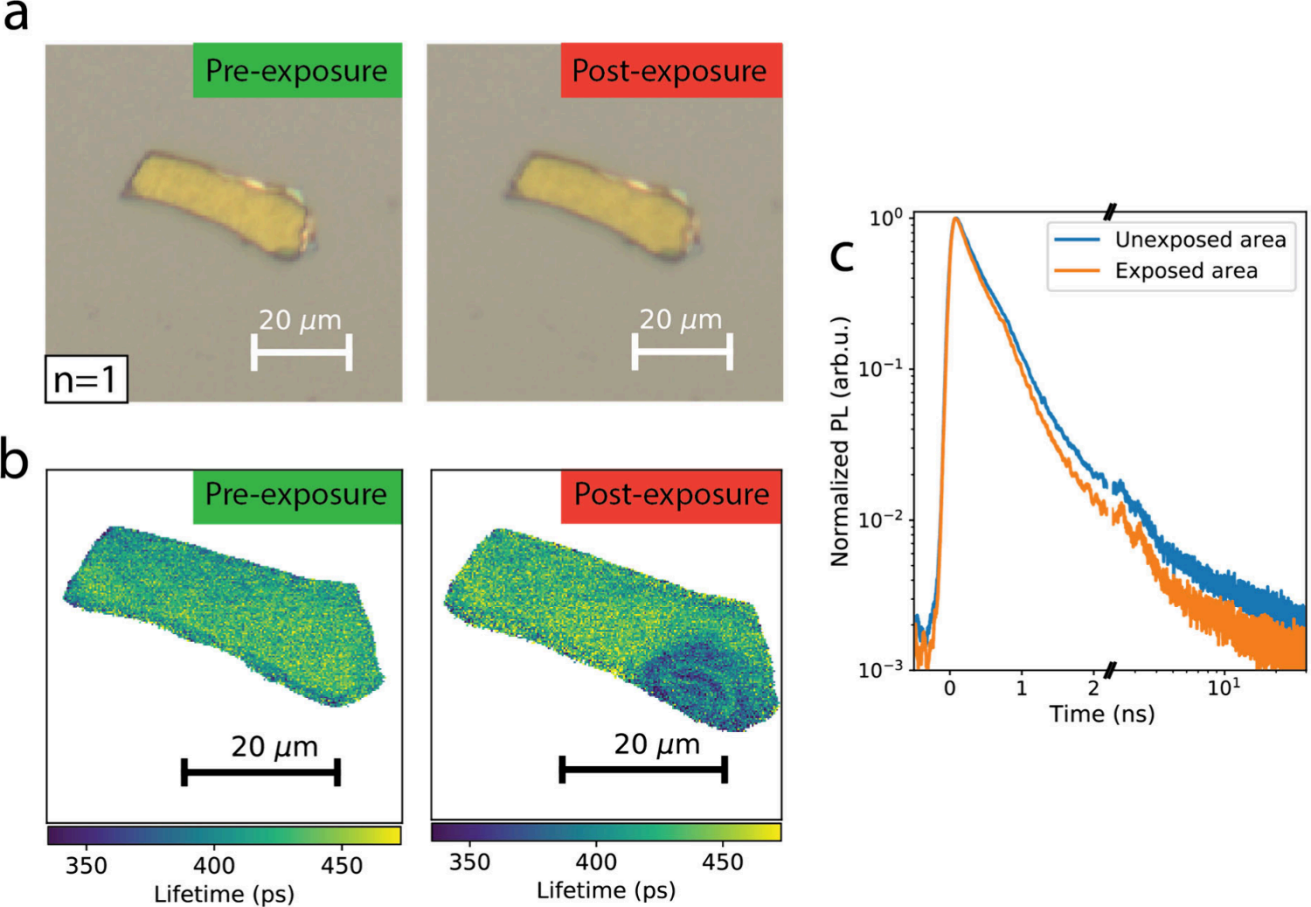
lifetime shortening in encapsulated flakes under illumination. (a) Optical images of an *n* = 1 2D perovskite flake before and after local photoexcitation, showing no signs of morphology changes. (b) PL lifetime images before and after photoexcitation of the same flake shown in (a), showing the reduction of the lifetime in the photoexcited area. (c) Time-dependence of the PL signal in the exposed area and in a pristine area, showing a shortening of the lifetime in the exposed area.

Having identified a clear difference between photodegradation processes affecting (quasi-) 2D perovskites in air and in nitrogen, we attempted to determine the relative contributions of oxygen and moisture to air-induced degradation. Figure 6a shows a comparison of the PL decay as a function of the incident dose for *n* = 1 flakes photoexcited with the same laser power under different environmental conditions. While nitrogen encapsulation leads to a slower PL decay, repeating the same encapsulation procedure in dry air (12.1% relative humidity) did not show enhanced stability compared to that of the unencapsulated sample (photoexcited at 55% relative humidity). This observation suggests that oxygen plays a dominant role in triggering the rapid photodegradation of the material, although the role of water cannot be fully excluded. At the same time, the result indicates that the confinement of volatile degradation products in the encapsulation volume does not significantly affect the photostability of the flakes.

**Figure 6:**
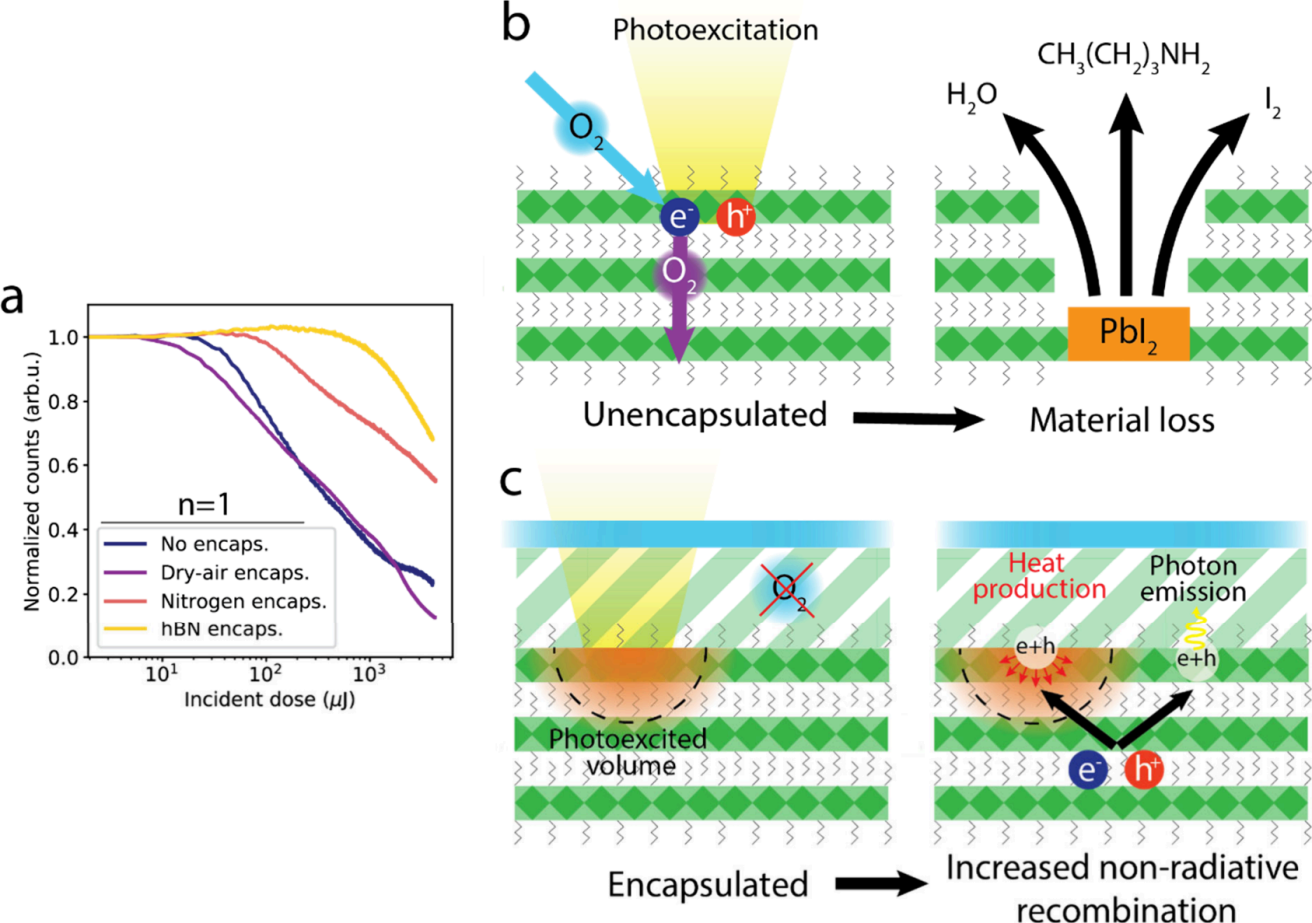
Passivation approaches and degradation mechanisms. (a) Comparison of PL decay traces obtained for *n* = 1 flakes in different environmental conditions, photoexcited with the same laser fluence. The comparison shows that stacking an hBN flake on top of the 2D perovskite flake and then encapsulating it in nitrogen leads to the highest photostability, while encapsulation in dry air (12.1% RH) does not improve the stability of the flakes. (b) Schematic representation of the proposed reaction mechanism in unencapsulated flakes, involving the formation of O_2_^–^ species that react with the perovskite structure, inducing its decomposition. (c) Schematic representation of the photoinduced increase in nonradiative recombination proposed for the encapsulated flakes.

Analogous to the degradation pathways proposed for 3D perovskites,^30^ we speculate that the photoinduced degradation of (quasi-) 2D perovskites follows the reaction
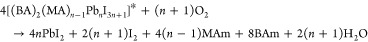
2with MAm and BAm indicating methylamine (CH_3_NH_2_) and butylamine (CH_3_(CH_2_)_3_NH_2_) species, MA and BA indicating methylammonium (CH_3_NH_3_^+^) and butylammonium (CH_3_(CH_2_)_3_NH_3_^+^) cations, and the asterisk indicating that the (quasi-) 2D perovskite material is in the photoexcited state. The reaction, schematically depicted in Figure 6b, involves the capture of a photoexcited electron by an oxygen molecule, leading to the formation of a reactive O_2_^–^ species and its diffusion into the material. Upon exposure to O_2_^–^, the (quasi-) 2D perovskite decomposes, releasing volatile species (H_2_O, I_2_, MAm, and BAm) and leaving behind PbI_2_.

In the absence of oxygen, the reaction described in eq 2 cannot happen. Consequently, photoexcitation under nitrogen leads mainly to an increase in nonradiative recombination channels, represented schematically in Figure 6c. Increased nonradiative recombination could be caused by carrier trapping at undercoordinated surface sites^31^ or by the accumulation of mobile ions.^32,33^ Despite the reduced severity of light-induced changes in the encapsulated flakes, further prevention of PL quenching might be the key to unlocking the full potential of these materials for spectroscopic investigations and device applications. To increase the photostability upon encapsulation, we stacked a flake of hexagonal boron nitride (hBN) on top of an *n* = 1 flake before encapsulating it in nitrogen (Figure S11), with the aim to simultaneously decrease the diffusion of residual oxygen into the flake and to passivate surface states.^22^ The resulting PL decay upon photoexcitation (hBN passivation in Figure 6a) shows a remarkable stability increase, as the unencapsulated flake reaches the final PL intensity of the hBN-passivated flake after being exposed to a 30× lower incident dose. These results demonstrate the need for synergistic passivation strategies to prevent the degradation processes affecting these materials.

In conclusion, we have shown the presence of strong photodegradation processes affecting *n* ≤ 4 (quasi-) 2D perovskites. Moreover, we have revealed the importance of oxygen for the degradation, proposing a reaction pathway and testing passivation strategies to suppress photodegradation. These results emphasize that the increased air-stability observed in 2D/3D solar cells should not be attributed to the intrinsic stability of quasi-2D perovskites and that a detailed understanding of the environment in which these materials are placed is required to rationalize their evolution upon illumination.

## Data Availability

The data that support the findings of this study are available from the corresponding author upon reasonable request.

## Supporting Information Available

Method section, additional figures, description of FTIR analysis, and description of photoluminescence lifetime fitting (PDF)
