# Supplementary material for: Atmospheric Exposure Triggers Light-Induced Degradation in 2D Lead-Halide Perovskites
Source: ACS Energy Lett. 2024 Nov 7;9(12):5771–9. doi: 10.1021/acsenergylett.4c02300 (PMC11650760; doi:10.1021/acsenergylett.4c02300)
Supplement: Supplementary file 1 — nz4c02300_si_001.pdf [file nz4c02300_si_001.pdf]

Atmospheric exposure triggers light-induced degradation in 2D lead-halide perovskites

Gianluca Grimaldi[1,2], Imme Schuringa[1], Jaco J. Geuchies[3], Susan A. Rigter[1,4], Tom Hoekstra[4], Jan Versluis[1], Juanita Hidalgo[5], Juan-Pablo Correa-Baena[5], Jorik van de Groep[4], Heejae Kim[3,6], Mischa Bonn[3], Bruno Ehrler[1*]

[1]Center for Nanophotonics, AMOLF, Science Park 104, 1098 XG Amsterdam, The Netherlands

[2]Optoelectronics section, Cavendish Laboratory, University of Cambridge, Cambridge, CB2 1TN, U.K.

[3]Department of Molecular Spectroscopy, Max Planck Institute for Polymer Research, 55128 Mainz, Germany

[4]Institute of Physics, University of Amsterdam, Science Park 904, Amsterdam, 1098 XH The Netherlands

[5]School of Materials Science and Engineering, Georgia Institute of Technology, Atlanta, Georgia 30332, United States

[6]Current address: Pohang University of Science and Technology (POSTECH), 77 Cheongam-ro, Nam-gu, Pohang-si, Gyeongsangbuk-do, South Korea

Methods

Sample fabrication

PbO (>99.9%), hydroiodic acid (57% w/w in water), hypophosphorous acid (H_3PO_2, 50% w/w in water), n-butylamine (99.5%) were purchased from Sigma-Aldrich. Methylammonium chloride (low water content) was bought from TCI. All chemicals were used without further purification.

The synthesis of cm-sized crystal flakes was done at a liquid-air interface, based on an adjusted protocol by Wang *et al*.[32] For the exact amounts, see tables S1-S4 in the Supporting Information. We mixed PbO in HI (57% w/w solution in water) and H_3PO_2 (50% w/w solution in water) in a 20mL glass vial. The mixture was heated to 80°C, at which point the PbO dissolved and a clear yellow solution is obtained. The thermocouple that controls the hot-plate temperature was always inserted in a 20 mL glass vial filled with the same volume of water as the perovskite precursor solutions.

Next to this, a solution of n-butylamine (nBAm) and methylammonium (MA) chloride in HI was prepared while cooling the vial, holding the nBAm and MA, in an icebath. Care should be taken to add the HI slowly, as the reaction is very exothermic. This nBAm/MA solution is dropwise added to the Pb-containing solution. Upon mixing, some small crystallites are formed, which dissolve rapidly.

Depending on the layer thickness that is formed, crystal growth is done either by lowering the temperature from 80°C to 70°C for n=1 and n=2, and crystal growth takes 10-30 minutes. For n=3 and n=4, we used a temperature controller (JKEM, controller type T), with a glass-coated probe inserted in a vial filled with water on the same hot plate, and reduced the temperature by 2°C per hour until nucleation of a crystal at the liquid-air interface, at which point the temperature was kept constant (usually between 55-65°C).

After the growth of the crystals at the liquid-air interface, the crystal is scooped gently off the interface with Teflon tweezers, placed on a plastic lid, and dried in a vacuum oven for 5 hours. After drying, the samples are stored in a nitrogen-filled glovebox until further use. Note that the overall crystal thickness varies from 10-20 micrometers for n=1 (making them very fragile) up to 200 micrometers for n=4.

The centimeter-sized (quasi-) 2D perovskite crystals were exfoliated inside a nitrogen-filled glovebox. The procedure consisted in sandwiching a piece of crystal between two pieces of Kapton tape, and then stripping them apart, resulting in the adhesion of a crystal flake to each of the two pieces. The procedure is repeated by taking one of the two Kapton tape pieces, pressing it to a fresh piece of Kapton tape, and stripping the two pieces. After repeating the stripping procedure three times, the resulting piece of Kapton tape is pressed to a piece of PDMS gel (Gel-Pak GEL-FILM, PF type) and peeled off with a rapid movement, leading to the transfer of (quasi-) 2D perovskite flakes to the gel. The piece of gel is then pressed on a glass substrate and slowly peeled off, resulting in flake transfer to the glass. Inspection of the freshly deposited crystals under the optical microscope shows that the PDMS has not left behind noticeable residues on the crystals.

For the 2D encapsulation, we exfoliated millimeter-sized hBN crystals in ambient conditions (relative humidity <20%) using Nitto SPV-224 tape. After six exfoliation steps, the tape was pressed onto a PDMS stamp (Gel-Pak GEL-FILM, WF type), and peeled rapidly to transfer hBN flakes to the stamp. Upon identification of a suitable multilayer hBN flake, we mounted the stamp in a custom transfer stage integrated with an optical microscope (WITec). The stamp was manually aligned over the target n=1 perovskite flake, and henceforth brought into contact. Finally, we slowly peeled the stamp, leaving behind the hBN capping layer on top of the perovskite target.

PL microscopy measurements

Local photoexcitation of (quasi-) 2D perovskite flakes and before/after photoexcitation imaging of the flakes was performed in a PL microscope setup (WITec), using a 405 nm laser source and imaging through a 10x objective. Photoluminescence collected by the objective is coupled into a glass fiber and sent to a spectrograph, allowing to record the spectral shape of the emission from the excited spot. To obtain intensity traces, the PL spectra for different *n*-values where integrated in a 20 nm-wide spectral range, centered at 520 nm (n=1), 580 nm (n=2), 618 nm (n=3), 653 nm (n=4).

Time-resolved PL imaging

Time-resolved PL measurements were performed in a confocal geometry, sending the output of a pulsed laser diode (485 nm laser diode, controlled by a PicoQuant PDL 828 "Sepia II" laser driver) on the sample through a water-immersion microscope objective, and sending the photoluminescence to an APD (MPD, connected to a PicoQuant HydraHarp 400 TCSPC system). A repetition rate of 20 MHz is used, and the data is binned in time with a resolution of 1 ps. The sample is mounted on a piezo stage, allowing automated x-y scanning and recording of lifetime information as a function of the position on the sample. Lifetime maps are obtained fitting the time-dependence of the PL signal obtained at each stage position with the convolution of a Gaussian Instrument Response Function (IRF, 44 ps standard deviation) with a single exponential decay (See Figure S9 in Supporting Information).

SEM/EDX measurements

SEM images were obtained with a FEI Verios 460 SEM. EDS measurements were performed in a FEI Verios 460 SEM, equipped with an Oxford Instruments EDX detector, at 15 kV and 0.8 nA.

FTIR measurements

The FTIR measurements were performed in a Bruker Vertex 80v FTIR spectrometer (Bruker), equipped with a liquid-nitrogen-cooled mercury-cadmium telluride (MCT) detector, and coupled to a Hyperion 3000 FT-IR microscope (Bruker). The spectra were recorded under a nitrogen atmosphere at a wavelength resolution of 4 cm^{-1}. For every spectrum, 128 scans were averaged. All measurements were performed in transmission mode on flakes deposited on silicon substrates. IR-blocking windows in the detection path were used to restrict the measured area to the photoexcited spot area. The reported spectra were corrected for the absorption of the bare silicon substrate. Furthermore, a background induced by interference effect was subtracted from the data (See Supporting Information, Figure S5). The FTIR measurements on photoexcited areas for the unencapsulated sample (Figure 2d) and for the encapsulated sample (Figure 3d) are performed on spots photoexcited with the same fluence of $1.5*10^{19}$ photons/cm^2.

XRF measurements

Nanoprobe X-ray fluorescence (nano-XRF) measurements were performed at the 2-IDD beamline of the Advanced Photon Source at Argonne National Laboratory. Nano-XRF was measured under a helium atmosphere, with a focused X-ray beam of 250 nm full-width-half-maximum, set at 13.8 keV. The maps were scanned with a step size of 250 nm and a full fluorescence spectrum was collected at each measurement point. Fitting, deconvolution and removal of the background was performed with the MAPS software.[33] Pb and I fluorescence signals were quantified based on NIST thin-film standards SRM 1832 and 1833 for calibration on the elemental concentrations.

Precursor amounts for the synthesis of $(BA)_2(MA)_{n-1}Pb_nI_{3n+1}$ single crystals

Table S1, amount of precursors used in the synthesis of the $(BA)_2PbI_4$ crystals (n=1)

**n=1**	**Chemical**	**Amount (mmol)**	**Amount (mg)**	**Amount (mL)**
**PbI solution**	PbO powder	5	1116	
	57% w/w acqueous HI solution	38		5
	50% (w/w I think) acqueous H_3PO_2 solution	7.75		0.85
**n-$CH_3(CH_2)_3NH_3I$ solution**	n-$CH_3(CH_2)_3NH_2$ (liquid)	5		0.462
	57% w/w acqueous HI solution	19		2.5

Table S2, amount of precursors used in the synthesis of the $(BA)_2(MA)Pb_2I_7$ crystals (n=2)

**n=2**	**Chemical**	**Amount (mmol)**	**Amount (mg)**	**Amount (mL)**
**PbI solution**	PbO powder	5	1116	
	57% w/w acqueous HI solution	38		5
	50% (w/w I think) acqueous H_3PO_2 solution	7.75		0.85
**n-$CH_3(CH_2)_3NH_3I$ solution**	CH_3NH_3Cl	2.5	169	
	n-$CH_3(CH_2)_3NH_2$ (liquid)	7		0.694
	57% w/w acqueous HI solution	19		2.5

Table S3, amount of precursors used in the synthesis of the $(BA)_2(MA)_2Pb_3I_{10}$ crystals (n=3)

**n=3**	**Chemical**	**Amount (mmol)**	**Amount (mg)**	**Amount (mL)**
**PbI solution**	PbO powder	5	1116	
	57% w/w acqueous HI solution	38		5
	50% (w/w I think) acqueous H_3PO_2 solution	7.75		0.85
**n-$CH_3(CH_2)_3NH_3I$ solution**	CH_3NH_3Cl	3.33	225	

	n-$CH_3(CH_2)_3NH_2$ (liquid)	1.67		0.164
	57% w/w acqueous HI solution	19		2.5

Table S4, amount of precursors used in the synthesis of the $(BA)_2(MA)_3Pb_4I_{13}$ crystals (n=4)

**n=4**	**Chemical**	**Amount (mmol)**	**Amount (mg)**	**Amount (mL)**
**PbI solution**	PbO powder	5	1116	
	57% w/w acqueous HI solution	38		5
	50% (w/w I think) acqueous H_3PO_2 solution	7.75		0.85
**n-$CH_3(CH_2)_3NH_3I$ solution**	CH_3NH_3Cl	3.75	253.5	
	n-$CH_3(CH_2)_3NH_2$ (liquid)	1.25		0.124
	57% w/w acqueous HI solution	19		2.5

Photoluminescence spectra of $(BA)_2(MA)_{n-1}Pb_nI_{3n+1}$ flakes

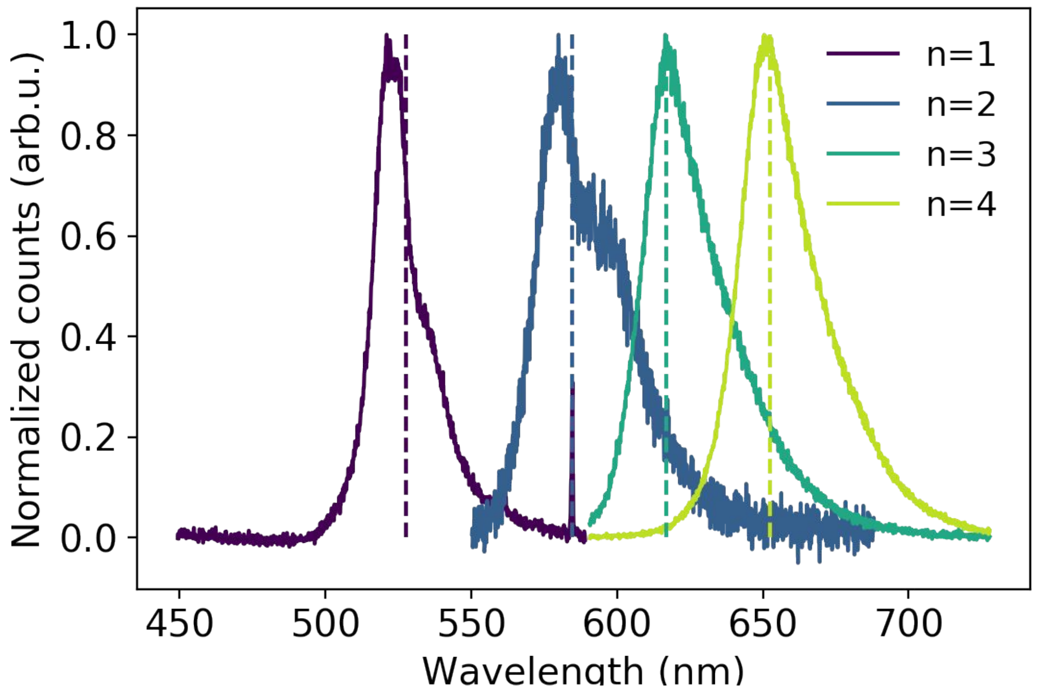

Figure S1, normalized photoluminescence spectra of the unencapsulated $(BA)_2(MA)_{n-1}Pb_nI_{3n+1}$ flakes measured in Figure 2a and Figure 4.

Figure S1 shows photoluminescence spectra obtained from the $(BA)_2(MA)_{n-1}Pb_nI_{3n+1}$ flakes measured in Figure 2a and Figure 4, photoexcited with 405 nm laser light at an incident photon fluence of $6*10^{18}$ photons/(cm^2*s).

Long range morphological changes in unencapsulated flakes

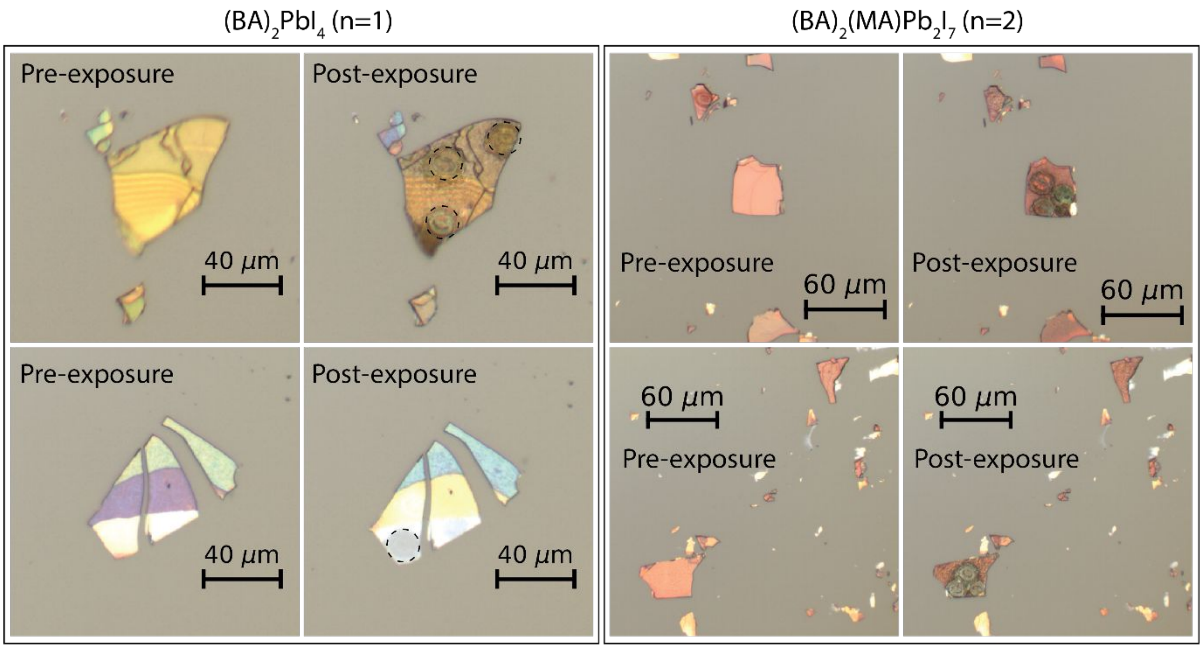

Figure S2, optical microscope images of unencapsulated $(BA)_2PbI_4$ (n=1) and $(BA)_2(MA)Pb_2I_7$ (n=2) flakes before and after local photoexcitation. The images show color changes after photoexcitation affecting unexcited regions of the photoexcited flakes and extending to neighbouring flakes.

Energy Dispersive X-ray Analysis (EDX) of a $(BA)_2(MA)Pb_2I_7$ flake

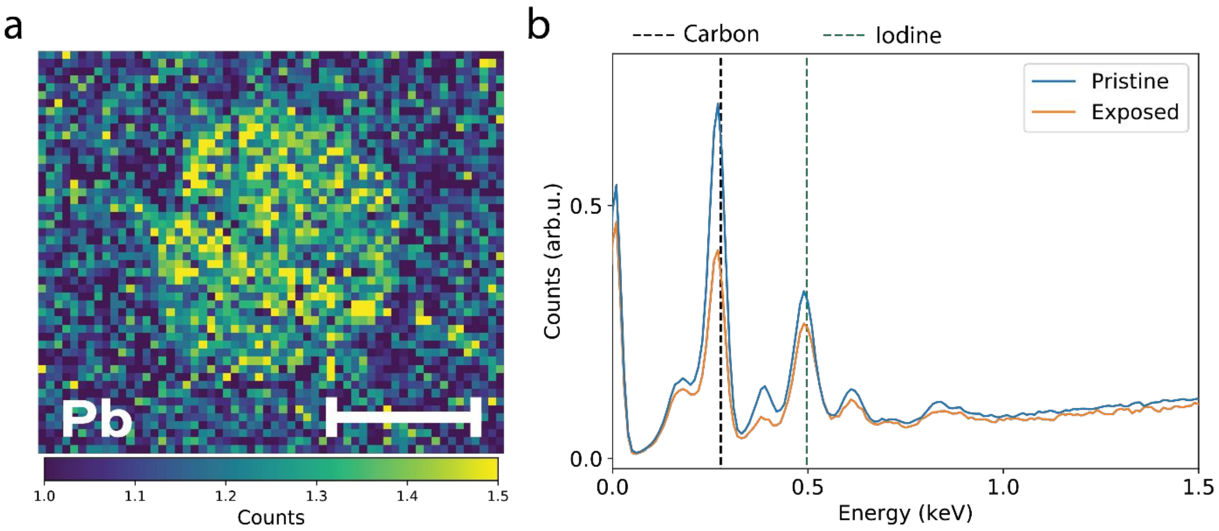

3

Figure S4, EDX measurements on an unencapsulated $(BA)_2(MA)Pb_2I_7$ flake (n=2). a) color map showing the intensity of an EDX peak associated with Pb content around an exposed area, highlighting an apparent increase in Pb signal in the exposed area. **b)** portion of the EDX energy spectrum containing a peak associated with carbon (black dotted line), showing a strong reduction in intensity in the measurement on the exposed area (orange line).

Figure S3a shows a colormap of the intensity of an EDX associated with lead, measured around an exposed spot. The measurement shows an increase in the signal in the photoexcited spot, likely due to an increase in the density of lead in the degradation product.

Figure S3b shows the low energy portion of EDX spectra measured on a pristine (blue) and exposed (orange) region of a $(BA)_2(MA)Pb_2I_7$ flake, highlighting the position of the peak associated with carbon (0.277 keV) and a peak associated with iodine (0.497 keV). The intensity of the carbon peak is strongly reduced in the exposed area, suggesting a desorption of the organic components following photoexcitation.

X-Ray Fluorescence (XRF) measurements on a photoexcited $(BA)_2(MA)_2Pb_3I_{10}$ (n=3) flake

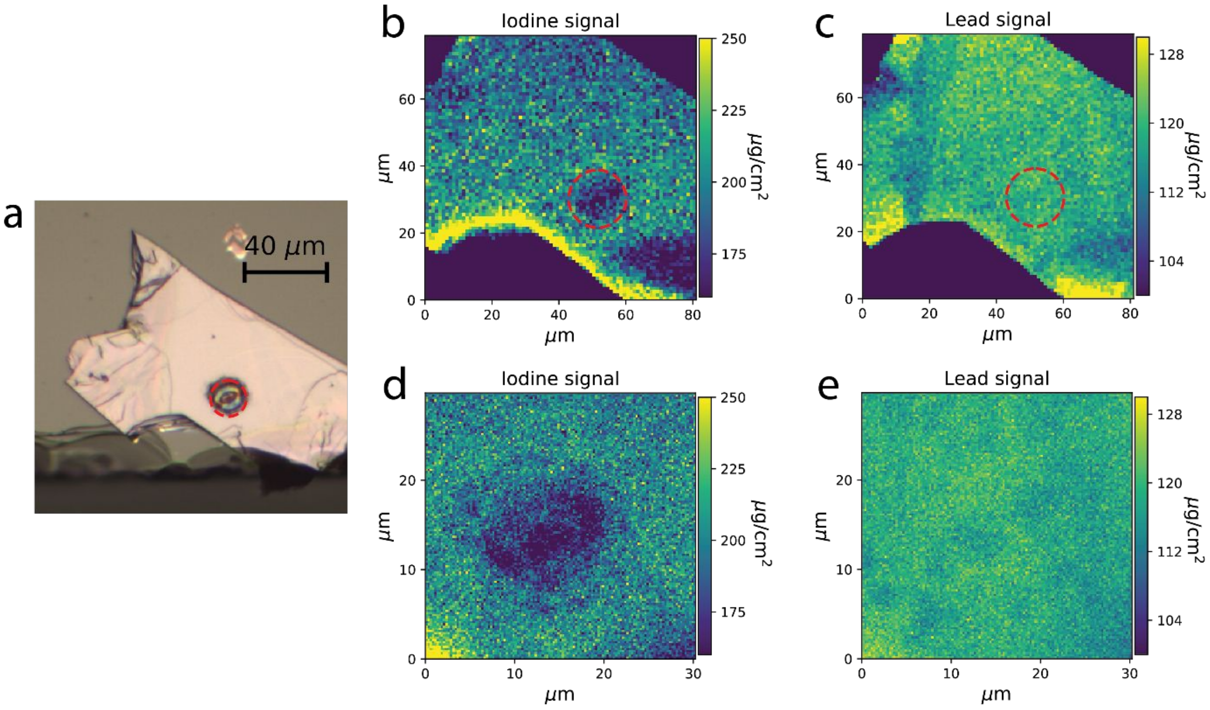

Figure S5, XRF measurements on an unencapsulated n=3 flake. a) optical image of the flake measured in XRF, highlighting the location of the photoexcited spot (red dotted line). **b,c)** colormaps showing the area density of the iodine (b) and the lead (c) component in the area around the photoexcited spot, extracted from the XRF data. The location of the photoexcited spot is highlighted by the red dotted line. Scan performed with a 1 μm step-size. **d,e)** colormaps showing the results of an XRF scan performed with smaller step-size (250 nm), focusing on a smaller area around the photoexcited spot.

Analysis of FTIR spectra

The FTIR measurements on the flakes were performed measuring the intensity of transmitted IR radiation on selected spots on the flakes. The intensity of transmitted IR radiation on the bare glass substrate is used as a reference to calculate the absorbance of the flakes:

$$A_{flake} = -\log_{10}\left({I_{flake}}/{I_{ref}}\right)$$

where I_{flake} is the intensity on the flake and I_{ref} the intensity on a bare area of the substrate.

Despite being corrected for the absorption of the glass substrate, absorbance measurements performed on the perovskite flakes are affected by signal oscillations as a function of the wavenumber (Figure S5a and S5c) likely due to light interference, as the sub-micrometer thickness of the flakes is comparable to half of the wavelength in the medium. Therefore, thickness variations in the flakes, such as those expected on morphology-altered photoexcited spots, will induce variations in the interference pattern.

In contrast to the slow variations of the interference pattern as a function of frequency, absorption peaks can be identified in the FTIR spectrum by their sharp frequency dependence and a lack of shift in frequency when changing location on the flake. Therefore, to compare the amplitude of the sharp peaks observed around 1500 cm^{-1} for different spots on the same flake, we assume a linear change in the background in the 1400 – 1650 cm^{-1} range and approximate the background with a line taking the value of the experimental data at 1400 and 1650 cm^{-1}. Figures S5b and S5d show the result of subtracting the linear background from the FTIR signal, highlighting the changes in the amplitude of the FTIR peaks above the background.

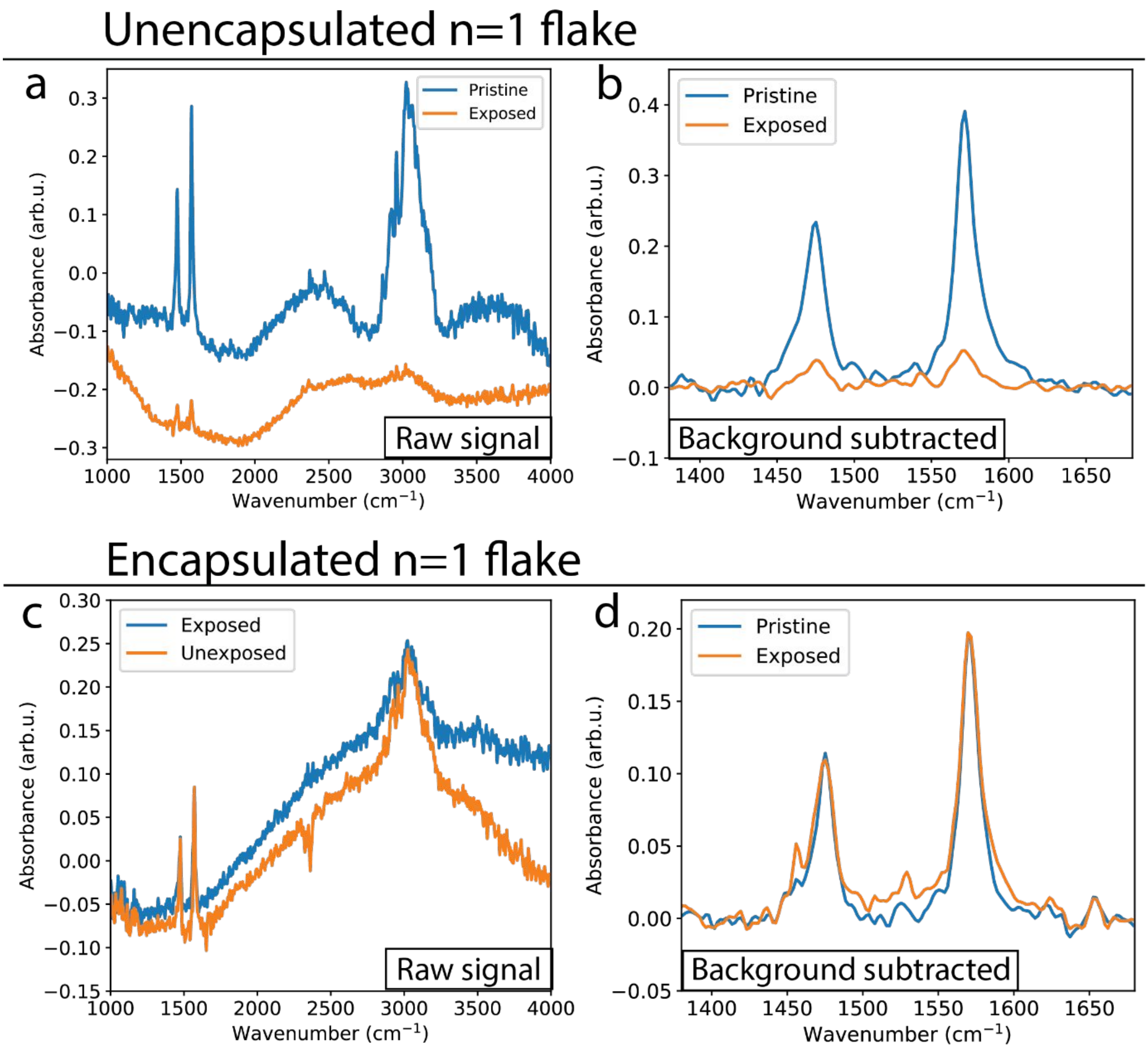

Figure S6, background subtraction from FTIR measurements. a) FTIR absorption measurements on an n=1 flake photoexcited in air (without encapsulation), corrected for the absorbance of glass. **b)** Absorption traces shown in panel a after background subtraction. **c)** FTIR absorption measurements on a n=1 flake photoexcited in N_2 (with encapsulation), corrected for the absorbance of glass. **d)** Absorption traces shown in panel c after background subtraction.

FTIR measurements on a $(BA)_2(MA)Pb_2I_7$ (n=2) flake photoexcited in N_2

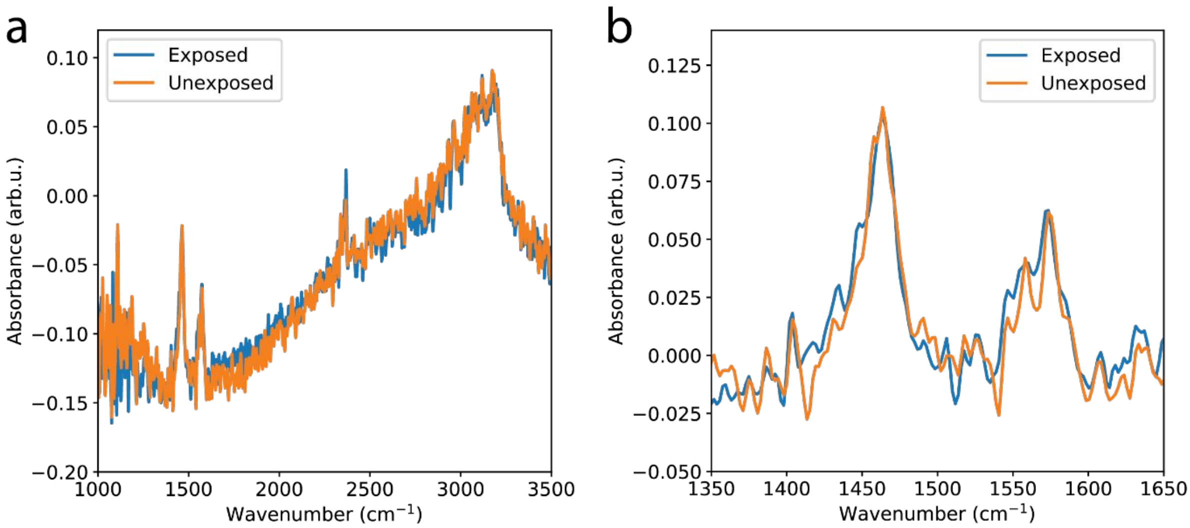

Figure S7, a) FTIR measurements on a (BA)2(MA)Pb2I7 (n=2) flake photoexcited in N2, corrected for the absorbance of glass. **b)** Absorption traces shown in panel a after background subtraction.

Simulation of the IR absorption spectrum of butylammonium

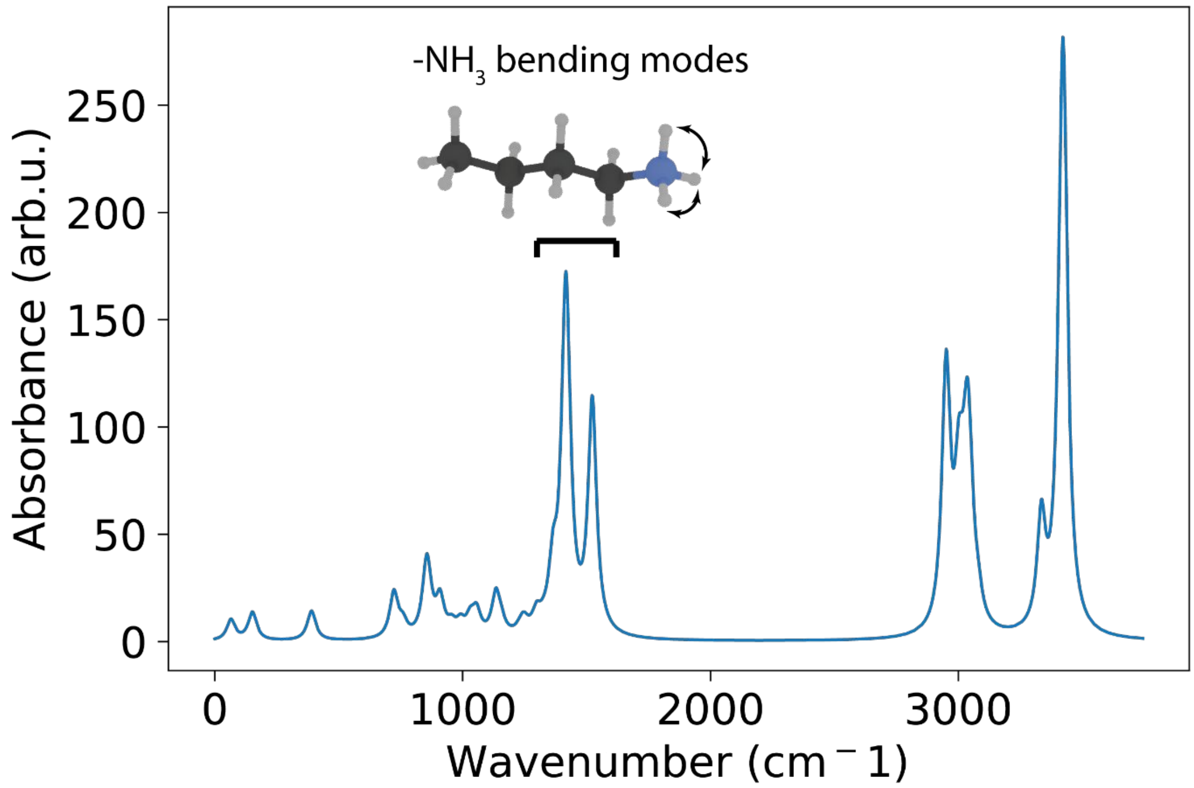

Figure S8, IR absorption spectrum of the butylammonium cation, simulated with the ORCA quantum chemistry program[1], using the libint2 library for the evaluation of molecular integrals[2]. The strongest absorption peaks in the 1400-1600 cm^{-1} range correspond to bending modes of the –NH_3 functional group in the butylammonium molecule. The simulated spectrum is in qualitative agreement with the FTIR measurement on n=1 2D perovskite flakes shown in Figure 2d and 3d, showing two sharp peaks in the 1400-1600 cm^{-1} range (1475 cm^{-1} and 1571 cm^{-1}).

Fractional decay of encapsulated and unencapsulated flakes

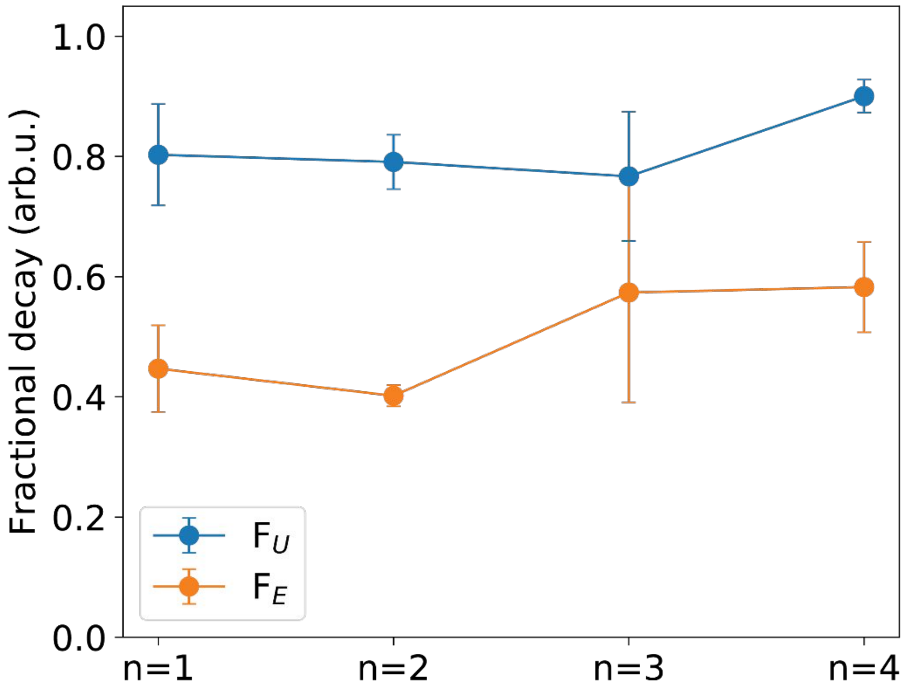

Figure S9, plot of the fractional decay FU and FE for the different n-values. Each data point is an average of the fractional decay measured for different laser fluences in the $2.2*10^{18}$ to $4.5*10^{19}$ photons/(s*cm^2) range, and error bars are given by the standard deviation of the fractional decay values.

Fitting the lifetime of photoluminescence of a $(BA)_2PbI_4$ flake

Assuming a single decay rate for the carrier population in a photoexcited $(BA)_2PbI_4$ flake, the decay of the PL signal can be described by a convolution of a gaussian Instrument Response Function (IRF) with an exponential decay multiplied by a step function:

$$f_{\text{signal}}(t) = Ae^{-\frac{(t-t_0)}{\tau}}\theta(t-t_0) \qquad \textbf{(eq. 1)}$$

$$f_{\text{IRF}}(t) = e^{-\frac{t^2}{2\sigma^2}} \qquad \textbf{(eq. 2)}$$

$$\left(f_{\text{signal}} * f_{\text{IRF}}\right)(t) = A\sigma\sqrt{\frac{\pi}{2}}e^{-\frac{t-t_0}{\tau}}e^{\frac{\sigma^2}{2\tau^2}}\left(1+\text{erf}\left(\frac{t-t_0}{\sqrt{2}\sigma}-\frac{\sigma}{\sqrt{2}\tau}\right)\right) \qquad \textbf{(eq. 3)}$$

The convolution can be fitted to the PL counts as a function of time summed over a large area on the flake, optimizing for the parameters A, σ, τ, and t_0 (Figure S9). The fit allows reliable extraction of σ and t_0, which can be kept fixed when fitting the time-dependence of the PL signal in each pixel (See Figure 5b). We note the presence of weak bumps in the decay traces, with one of them visible 1 ns after signal rise in Figure S10 and Figure 5, and an additional feature visible around 2-3 ns in Figure 5.

These features are likely due to the fact that the IRF is not perfectly Gaussian. These deviations, however, do not significantly change the fitted lifetimes.

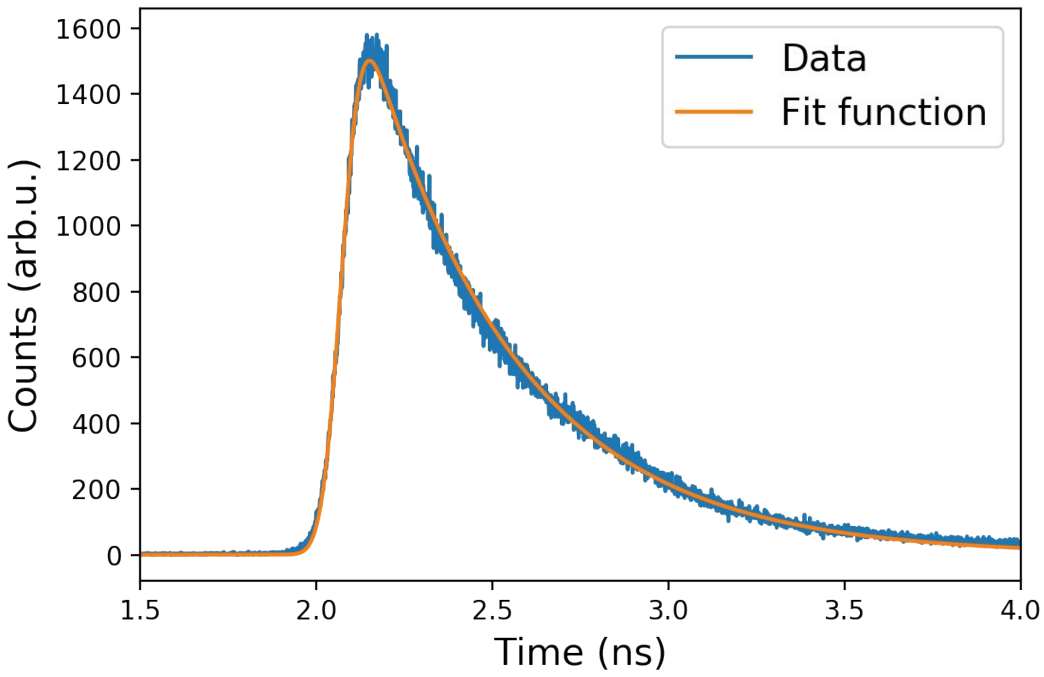

Figure S10, time dependence of the PL signal from the same n=1 flake shown in Figure 5, summed over an unexposed area on the flake (blue line). The orange line shows the fit function obtained fitting equation 3 to the data, optimizing for the parameters A, σ, τ, and t_0. The standard deviation σ for the Gaussian IRF function is determined to be 43.80 ± 0.9 ps.

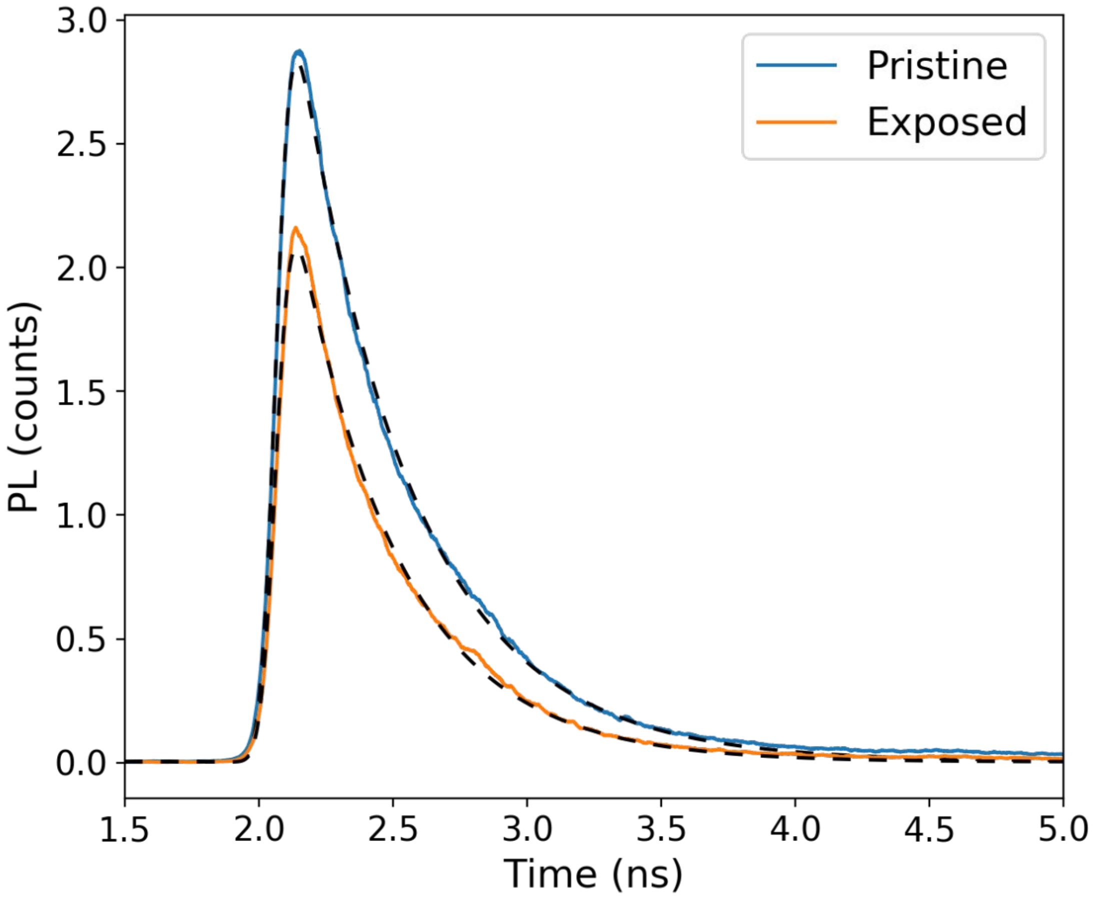

Figure S11, time dependence of the PL signal from the same n=1 flake shown in Figure 5, comparing the signal averaged over an unexposed area on the flake (blue line) and the signal averaged over an exposed area (orange). The black lines show the fit functions obtained fitting equation 3 to the data, optimizing for the parameters A, σ, τ, and t_0. The lifetime τ extracted from the fit has a value of 431.0 ± 0.2 ps for the pristine area and 388.9 ± 0.2 ps for the exposed area.

Hexagonal boron nitride (hBN) encapsulation of $(BA)_2PbI_4$ flake

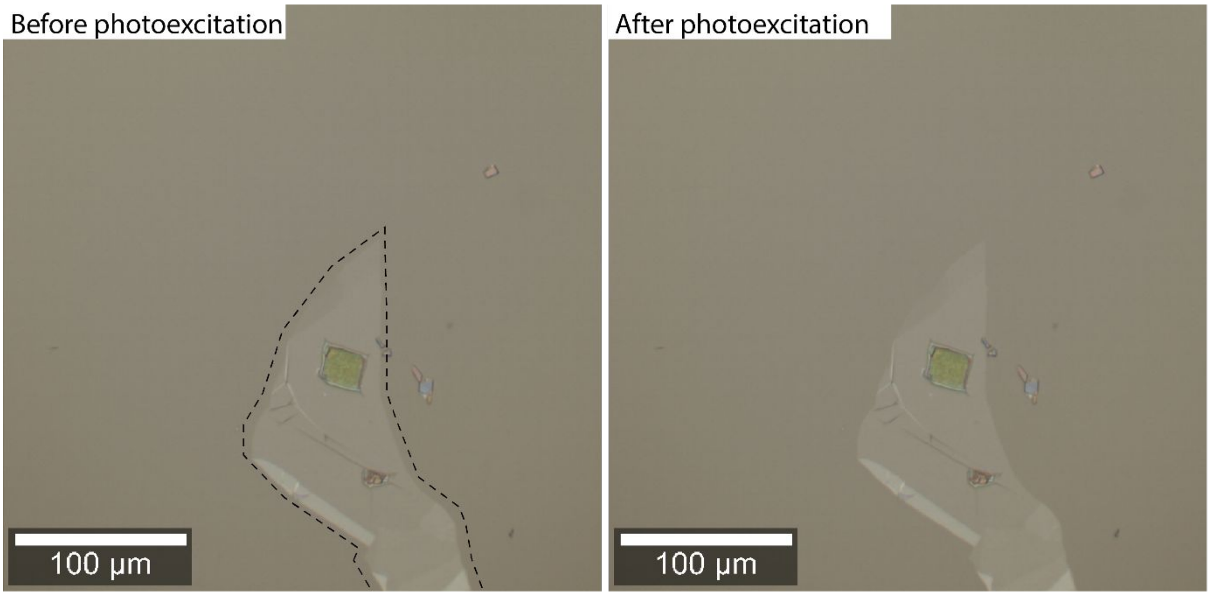

Figure S12, optical microscope images of a $(BA)_2PbI_4$ flake encapsulated with a layer of hexagonal boron nitride (hBN), showing the absence of morphology change after photoexcitation of the flake with 405nm laser light and an incoming fluence of $2.2*10^{18}$ photons/(s*cm^2).

References

(1) Neese, F. The ORCA Program System. *WIREs Computational Molecular Science* **2012**, *2* (1), 73–78.

(2) Valeev, E. F. Libint: A Library for the Evaluation of Molecular Integrals of Many-Body Operators over Gaussian Functions.
